# Chitin and Cellulose Processing in Low-Temperature Electron Beam Plasma †

**DOI:** 10.3390/molecules22111908

**Published:** 2017-11-06

**Authors:** Tatiana Vasilieva, Dmitry Chuhchin, Sergey Lopatin, Valery Varlamov, Andrey Sigarev, Michael Vasiliev

**Affiliations:** 1 Moscow Institute of Physics and Technology, Institutsky per., 9, Dolgoprudny, 141700 Moscow, Russia; aasigarev@mail.ru (A.S.); mnvasiliev2006@rambler.ru (M.V.); 2 Core Facility Center “Arktika”, Northern (Arctic) Federal University, Severnaya Dvina Emb., 17, 163002 Arkhangelsk, Russia; dimatsch@gmail.ru; 3 Federal State Institution, Federal Research Centre, Fundamentals of Biotechnology of RAS, Institute of Bioengineering, 60 let Oktjabrja pr-t, 7/1, 117312 Moscow, Russia; lopatin@biengi.ac.ru (S.L.); varlamov@biengi.ac.ru (V.V.)

**Keywords:** chitin, cellulose, oligosaccharides, low-temperature plasma, electron-beam plasma, plasma chemistry

## Abstract

Polysaccharide processing by means of low-temperature Electron Beam Plasma (EBP) is a promising alternative to the time-consuming and environmentally hazardous chemical hydrolysis in oligosaccharide production. The present paper considers mechanisms of the EBP-stimulated destruction of crab shell chitin, cellulose sulfate, and microcrystalline cellulose, as well as characterization of the produced oligosaccharides. The polysaccharide powders were treated in oxygen EBP for 1–20 min at 40 °C in a mixing reactor placed in the zone of the EBP generation. The chemical structure and molecular mass of the oligosaccharides were analyzed by size exclusion and the reversed phase chromatography, FTIR-spectroscopy, XRD-, and NMR-techniques. The EBP action on original polysaccharides reduces their crystallinity index and polymerization degree. Water-soluble products with lower molecular weight chitooligosaccharides (weight-average molecular mass, *M_w_* = 1000–2000 Da and polydispersity index 2.2) and cellulose oligosaccharides with polymerization degrees 3–10 were obtained. The ^1^H-NMR analysis revealed 25–40% deacetylation of the EBP-treated chitin and FTIR-spectroscopy detected an increase of carbonyl- and carboxyl-groups in the oligosaccharides produced. Possible reactions of β-1,4-glycosidic bonds’ destruction due to active oxygen species and high-energy electrons are given.

## 1. Introduction

Two natural renewable biopolymers, namely cellulose (linear chain of several hundreds to many thousands of β-1,4-linked d-glucose units) and chitin (linear heterocopolymer of β-1,4-linked 2-amino-2-deoxy-d-glucopyranose and 2-acet-amido-2-deoxy-d-glucopyranose units), are the most abundant polysaccharides on the earth. Cellulose, chitin, and chitosan derivatives are very promising for technological and industrial applications such as agriculture, microbiology, food processing, medicine, and the pulp and paper subsector [1,2,3,4,5,6].

Though the polymers possess unique properties (high biocompatibility with living tissues, biodegradability, ability to the complexation, low toxicity, etc.) they have limited applications in many industrial fields because of their insolubility in most solvents. For example, water-soluble low molecular weight chitooligosaccharides (COS) (less than 10 kDa) are usually required in medicine, pharmaceutics, and agriculture [5,6,7,8,9,10]. Low-molecular weight cellulose fragments are prospective substrates for microbiology since they can be converted into monosaccharides and a number of products, fermentable sugars for bio-ethanol synthesis via microbial fermentation among them [11].

Simple and relatively low-cost chemical hydrolysis in concentrated acids or alkalis at high temperatures is a conventional method. However, these techniques usually take several hours or even days and the processing equipment is damaged due to corrosion and neutralization procedures are required [12,13,14,15]. Moreover, toxic waste and environmental contamination are inherent in polysaccharide chemical processing. Another problem with the chemical methods is the formation of products with modified chemical structures. For instance, the chemical degradation of chitin can result in the formation of deacetylated COS but for further (bio)chemical reactions the stable *N*-acetyl groups available in the chitin structure are usually required [12,13].

Enzymes are often preferable to inorganic compounds with catalytic capacity because they are environmentally sustainable. However, the enzymatic hydrolysis of cellulose is often incomplete, and the products formed have a high polydispersity index [16].

Thus, the efforts towards the development of new economically feasible and environmentally friendly methods for polysaccharide degradation appear to be reasonable. The plasma-chemical technologies using non-equilibrium, low-temperature plasmas could be a promising alternative to the hydrolysis methods mentioned above. Our previous papers have shown that electron-beam plasma (EBP) can be applied for the effective and controllable destruction of chitosan, and high yields of water-soluble COS can be attained by optimizing the EBP-treatment procedure [17].

The aims of the present study were as follows:

- To study the EBP-processing of chitin and cellulose and to reveal the time dependence for the EBP-stimulated formation of water-soluble low molecular weight products (LMWP).
- To characterize the chemical structure and molecular mass of the produced LMWP.
- To suggest possible mechanisms of EBP action on polysaccharides.

## 2. Results

### 2.1. The EBPProcessing of Polysaccharides

**Figure 1:**
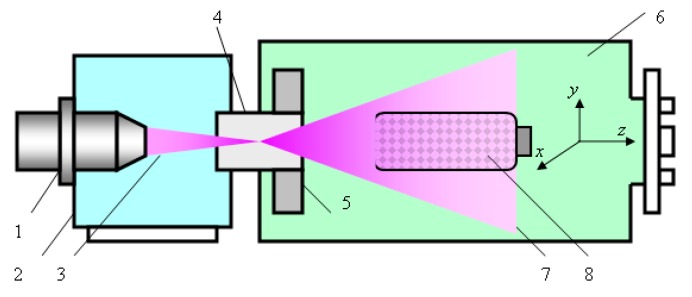
Electron-beam plasma-chemical reactor and scheme of the treatment procedure: 1—electron-beam gun; 2—high vacuum chamber; 3—electron beam; 4—injection window; 5—electromagnetic scanning system; 6—working chamber; 7—EBP cloud; 8—mixing device with polysaccharide powder to be treated.

Figure 1 illustrates the design and operation of the electron-beam plasma-chemical reactor (EBPR). The EBPR, its operation modes, and the optimization of the biomaterial treatment regimens, as well as the EBP properties, were described in detail in [18,19].

Briefly, the focused EB 3 generated by the electron-beam gun 1 that is located in the high vacuum chamber 2 is injected into the working chamber 6 filled with the plasma-generating gas through the injection window 4. In passing through the gas the EB is scattered in elastic collisions and the energy of fast electrons gradually diminishes in various inelastic interactions with the medium (ionization, excitation, dissociation). As a result, EBP cloud 7 is generated, all plasma parameters being functions of the *x*, *y* and *z* coordinates (*z* is the axis of the EB injection).

The electromagnetic scanning system 5 placed inside the working chamber near the injection window is able to deflect the injected EB axis in the *x* and *y* directions and, therefore, to control the spatial distribution of the plasma particles throughout the plasma bulk. The working chamber is preliminarily evacuated to pressure ~1 Pa and then filled with the plasma generating media—chemically pure oxygen (Sigma-Aldrich, Munich, Germany) up to 670 Pa.

**Figure 2:**
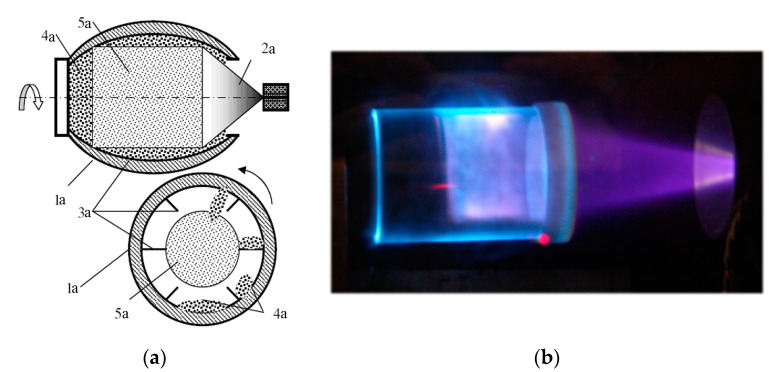
Polysaccharide powders processing in the mixing device: (**a**) Design of the mixing device for the EBP-treatment of polysaccharide powders: 1a—cylindrical quartz vessel; 2a—the EBP cloud; 3a—internal partitions; 4a—polysaccharides powder; 5a—aerosol reaction zone. (**b**) Chitin powder in the EBP reaction zone photo.

The polysaccharide powders were processed in a special mixing reactor 8. The device (Figure 2a) contains a cylindrical quartz vessel 1a with internal ribs 3a; it is equipped with a stepper motor rotating the vessel in various modes (continuous, intermittent, reverse, etc.). When the vessel rotates, the powder material to be treated (4a) is mixing. The device is placed inside the EBPR working chamber filled with the plasma generating gas at required pressure and the EB 2a is injected through the open end of the reactor. As a result the aerosol reaction zone 5a is formed inside the vessel. The treatment time duration (τ) of the polysaccharides varied within the range 1–20 min. To prevent the polysaccharides’ thermal destruction, they were processed at material temperature *T_s_* = 40 °C. The sample temperature during the treatment was monitored by an optical IR-pyrometer Optris LS (Optris GmbH, Portsmouth, NH, USA) or by a miniature thermo-sensor inserted into the reaction zone. The material temperature was controlled by varying the EB current.

### 2.2. The Characterization of the EBP-Treated Polysaccharides

#### 2.2.1. Molecular Mass Characterization

The original polysaccharides were water-insoluble and the EBP-treatment (τ = 1–10 min) increased their solubility in water and some other solvents due to the decrease of polymerization degree and the low molecular weight products (LMWP) formation. Table 1 illustrates the solubility changes of the EBP-treated cellulose sulfate in distilled water and 5% NaOH. The yield of soluble cellulose LMWP increased with the EBP-treatment time duration and reached 77.4% (for water-soluble LMWP) after a 10-min treatment. EBP-processing for 2 min significantly reduced the polymerization degree of the non-soluble fraction.

**Figure 3:**
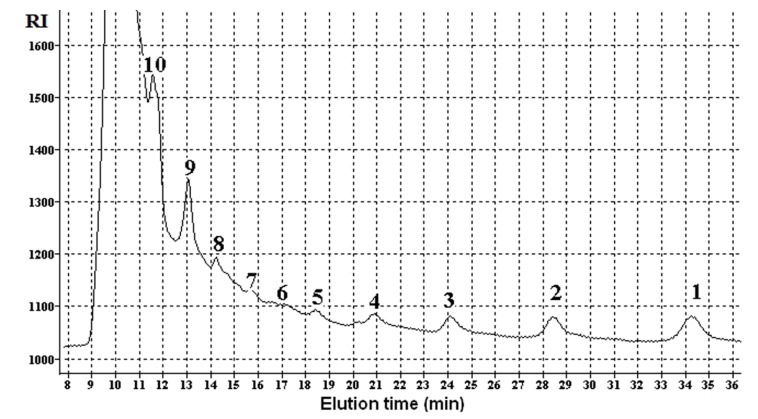
The ligand-exchange chromatogram of the water-soluble cellulose LMWP obtained due to the EBP-processing: 1—glucose; 2–10—LMWP with various polymerization degrees (the numbers correspond to the degree of polymerization); RI—refractive index.

**Figure 4:**
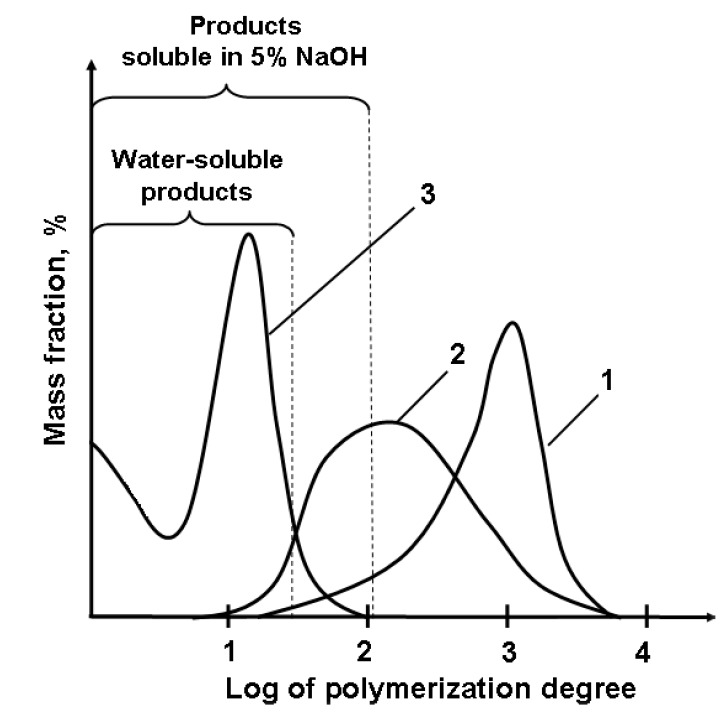
The molecular weight distribution of the products formed in the cellulose EBP-processing: 1—original cellulose; 2—cellulose treated in the EBP for 2 min; 3—cellulose treated in the EBP for 10 min.

The mixture of products with polymerization degrees varying from 10 and higher to glucose monomers were found in the extracts of the EBP-treated cellulose by the chromatography technique, the ion-exclusion column Rezex RSO-Oligosaccharide Ag^+^ (4%) (Phenomenex, Torrance, CA, USA) being used. The water-soluble LMWP have polymerization degrees mostly in the range of 3–10. Products with lower (up to glucose monomers) and higher molecular weights were also observed (Figure 3). At first, the concentration of LMWP drops with the decrease in polymerization degree down to 6 and then rises again. The molecular weight distribution of the formed products is presented in Figure 4. The LMWP formation with predominating tetramer was also confirmed by ^13^C-NMR analysis.

The exclusion chromatography of the EBP-treated chitin revealed formation of low-molecular weight chitooligosaccharides (COS) with weight-average molecular mass, *M_w_* = 1000–2000 Da and polydispersity index 2.2.

Since the formation of COS with *M_w_* = 800–2000 Da and polymerization degree varying from dimers to heptamers during the EBP-stimulated destruction of chitosan has been proved before [17], the same mechanism of the EBP-stimulated processing for all polysaccharides can be supposed.

#### 2.2.2. Main Features of the Polysaccharides EBP-Processing

Our present experiments revealed the dependence of the water-soluble LMWP yield on the treatment time and some other inherent features in chitin and cellulose EBP-processing were also found:

- At optimized treatment conditions (plasma generating media pressure, electron beam characteristics, the mixing device design)only 2 min were required to obtain 85% yield of low-molecular weight COS from original chitin powder [17]. The maximal yield of cellulose water-soluble LMWP (77.4%) was obtained after 10 min (Table 1).
- The threshold relationship between LMWP yield and the duration of the EBP-treatment. At optimized treatment conditions, chitin and cellulose destruction stopped when the processing time reached 7 and 15 min, respectively.

#### 2.2.3. Chemical Structure Characterization

A 30% decrease of the orderly phase in treated cellulose samples with respect to that of the original substance was found, while the content of amorphous fraction was increased. The cellulose crystallinity index (CI) was determined by the XRD technique. The amorphicity was found to increase after plasma-chemical treatment: the CI of modified cellulose was 76.4%, whereas the CI of the untreated substance was 86.4%. The same trend was shown for chitin. Preliminary experiments revealed the loss of CI in the following ranges: from 61.1% to 65.9% (original chitin) up to 50.9–55.2% after the EBP-treatment in oxygen-containing media.

**Figure 5:**
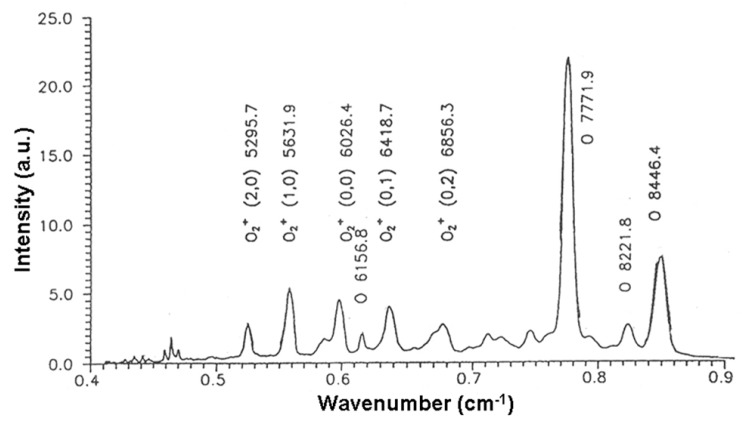
The optical spectrum of oxygen EBP in visible and NIR ranges; the peaks attributed to the different oxygen species are in accordance with [21,22].

**Figure 6:**
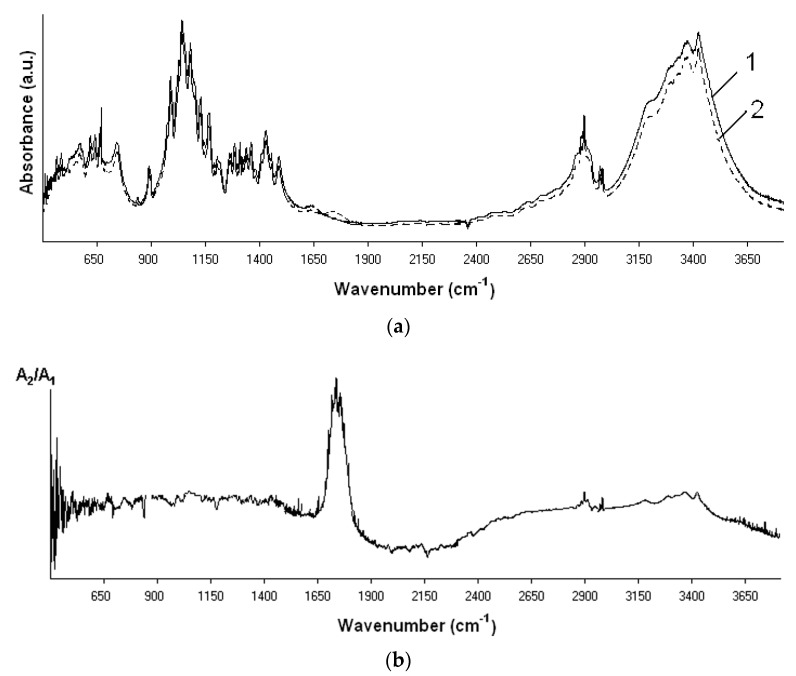
The IR-spectroscopy of cellulose: (**a**) The different IR-spectra of the cellulose before (1, A_1_) and after the EBP-treatment (2, A_2_) in oxygen media; (**b**) the ratio of the IR-spectra (A_2_/A_1_).

The low-temperature EBP is a source of several active oxygen species (O, O^•^, singlet oxygen, OH^•^, etc.) (Figure 5) that are produced in plasma chemical processes in very high concentrations (up to 10^10^–10^11^ cm^−3^) [18]. These particles can be responsible for both the destruction of polysaccharides and their oxidation. To reveal the oxidation changes in the polysaccharide molecules due to the treatment in the oxygen, FTIR-spectroscopy was applied. The IR-spectra analysis of cellulose showed intense absorbance at 1720–1750 cm^−1^ (Figure 6) that can be attributed to the significant increase of carbonyl C=O groups [20].

**Figure 7:**
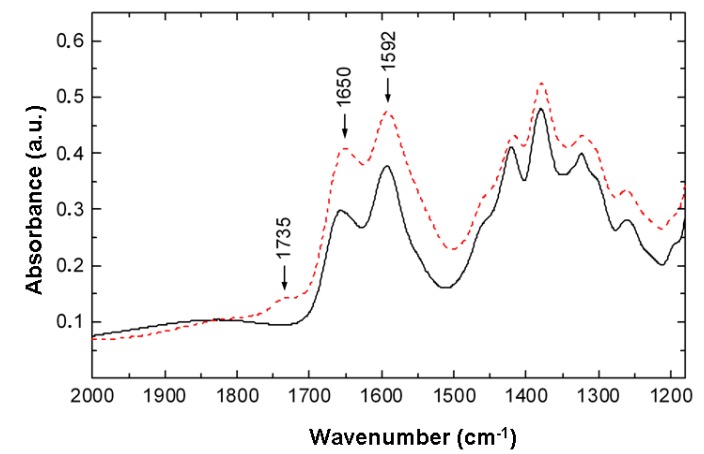
Partial absorbance spectra of the chitosan film before (solid black curve) and after (dashed red curve) the EBP-treatment in oxygen media in the range 1180–2000 cm^−1^.

Chemical changes in the structure of nitrogen-containing polysaccharides were studied using the EBP-treated chitosan films. Figure 6 shows the differences in the chitosan IR-spectra within the wave number range 1500–2000 cm^−1^. It is important that in the spectra of the treated chitosan (red curves in Figure 7) the weak band at 1735 cm^−1^ corresponding to the stretching vibrations of C=O groups in carboxyl groups (–COOH) is observed. The selective peak intensity (the difference between the absorbance values at the maximum and at the baseline level) for the band at 1650 cm^−1^ (corresponding to C=O stretching vibrations of Amide I groups) in the spectrum for the treated chitosan is approximately 20% higher than that in the absorbance spectrum of the original substance. Also there is a small decrease in the integral intensity of the bands at 1422, 1155, and 896 cm^−1^ in the range 1250–500 cm^−1^. Note that the bands at 1155 and 896 cm^−1^ correspond to β-1,4-glycosidic bonds [23,24,25]. So the analysis of the IR absorption spectra of the original and treated chitin showed that the EBP-treatment resulted in some increase of oxygen-containing carbonyl C=O and carboxyl –COOH groups and partial destruction of the β-1,4-glycosidic bonds.

To reveal the EBP effect changes on the chemical structure in chitin rings and to detect their possible oxidation, the monomeric substrate *N*-acetyl-d-glucosamine was treated in oxygen EBP and analyzed by IR-spectroscopy. The technique did not detect any oxidative changes in the EBP-treated *N*-acetyl-d-glucosamine.

**Figure 8:**
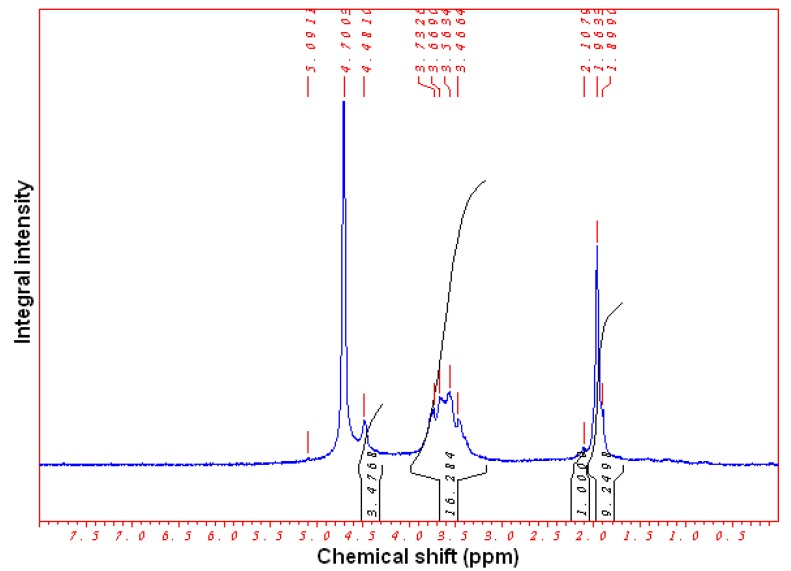
^1^H-NMR spectrum of the chitin treated in the oxygen for 15 min.

The reverse-phase chromatography (within the sensitivity of the technique) showed that the retention time of water-soluble COS produced in the EBP-treatment of chitin and retention time of model mixture of the individual *N*-acetylglucosamine oligomers was approximately the same [17]. More sensitive ^1^H-NMR analysis revealed 25–40% deacetylation of the EBP-processed chitin depending on the duration of the treatment (for τ = 5–10 min). The typical ^1^H-NMR spectrum of the EBP-treated chitin is shown in Figure 8 (τ = 15 min) and the example of the DD calculations is given below:

DA/DD = (10.2498/3)/(19.76/6) = 1.037, DA = 50.9 mol %, DD = 49.1 mol %(1)

(all comments and explanations are given in Section 4).

## 3. Discussion

### 3.1. Possible Mechanisms of the EBP-Action on Polysaccharides

The following EBP factors can affect the polysaccharides structure during their plasma chemical processing:

- Chemically active heavy particles of the EBP: excited molecules and atoms, ions, radicals (active oxygen species, for example);
- The fast electrons of the partially degraded EB that bombard the polysaccharide material;
- The secondary electrons of moderate energies (up to 0.01–1 keV) produced in the EBP due to ionization of the molecules of the plasma generating media;
- The EBP-radiation, especially UV one and X-ray (bremsstrahlung);
- Possible heating by direct electron bombardment and due to heat transfer between the plasma cloud and the sample.

Each of these factors is able to cause transformations of polymeric molecules, but the integral effect of the polysaccharides macromolecule modification is likely to be due to their joint action, i.e., synergism is expected to take place.

**Figure 9:**
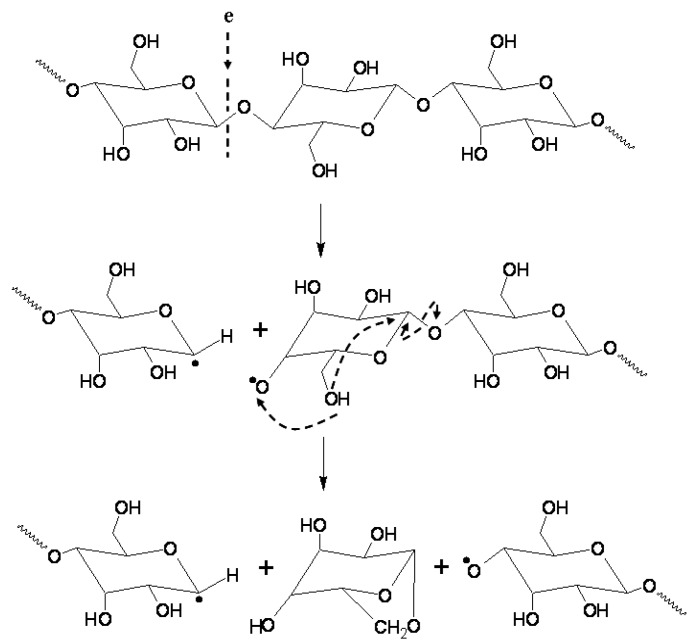
The scheme of cellulose degradation under action of high-energy electrons in the EBP.

The possible reaction of the fast electrons with polysaccharide chains results in the destruction of β-1,4-glycosidic bonds and dehydration with levoglucosan formation (Figure 9). The same conversion occurs during the pyrolysis of cellulose and other carbohydrates in a vacuum [26]. The increase of the radiation dose can stimulate further dehydration and production of low molecular weight organic compounds such as furfural and 5-hydroxymethylfurfural, furfuryl alcohol, enolactones, and various aromatic structures. These substances are markers of deep polysaccharide damage and their carbonization should be avoided. Under our experimental conditions these organic compounds were not detected in the EBP-treated samples. Thus the radiation and thermal polysaccharides damage due to high energy electrons has been minimized by proper selection of plasma generating gas pressure and the EB scanning mode [27,28].

**Figure 10:**
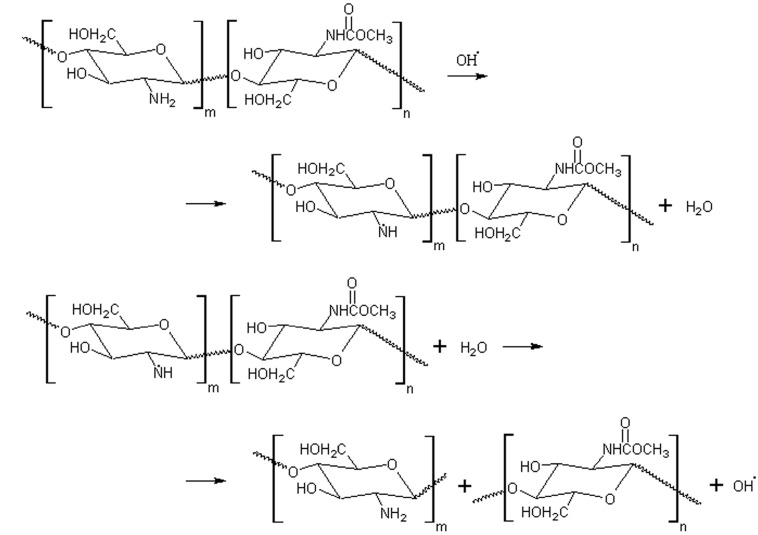
The scheme of chitin degradation under hydroxyl radical action in the EBP: the attack of OH radicals on the chitin –NH_2_ groups [32].

In our previous studies it was proven that plasma chemical processes predominate [27,28]. Chemically active oxygen particles break the β-1,4-glycosidic bound and decrease the polysaccharides’ molecular weight. These particles can abstract hydrogen atoms at all ring C–H bonds in carbohydrates except C-2 of *N*-acetyl hexosamine [29,30,31]. Figure 10 illustrates a possible degradation mechanism of chitin during its treatment in the EBP of water vapor [32]. The reaction involves the abstraction of a hydrogen atom with the generation of carbon-center radicals. Then the radicals at carbons that form glycosidic bonds undergo a β-scission reaction, resulting in the breakdown of polysaccharide chains [29,30,33]. The oxidation changes in the polysaccharides molecules with increase of carbonyl C=O and carboxyl –COOH groups after the treatment in the EBP of oxygen also support this hypothesis.

**Figure 11:**
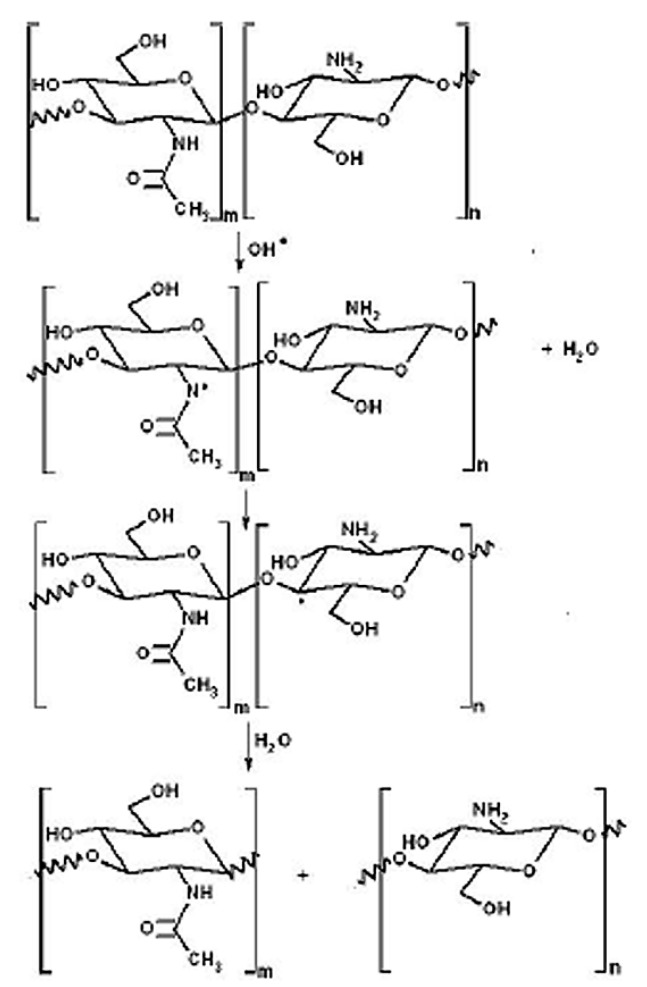
The scheme of chitin degradation under hydroxyl radical action in the EBP: the attack of OH radicals on the chitin –NH_2_ groups.

The second possible site of the OH radical action is the chitin *N*-acetyl group. This conversion was found for both amino acid derivatives like *N*-acetyl methionine [34] and glycosaaminoglycans (e.g., hyaluronan) [35,36] under oxygen-containing radicals and some other active oxygen species. The mechanism presented in Figure 11 assumes the C-4 carbon-centered radicals formation and subsequent scission reaction of these radicals leading to the cleavage of β-1,4-glycosidic bonds.

Simultaneously with the destruction of β-1,4-glycosidic bonds and the stimulation of oxidation reactions heavy plasma radicals and ions etch the polysaccharides crystals resulting in their CI loss and the amorphicity increase. The mechanisms responsible for the plasma-stimulated etching of organic polymers are well-known and described in details in [37,38,39]. These mechanisms, which are shown to be common for various polymers, explain the same CI decrease values and trends for both chitin and cellulose.

### 3.2. The Comparison of the EBP-Stimulated Polysaccharides Destruction with Respect to Acid-Catalyzed Hydrolysis

The comparison of the EBP-stimulated polysaccharides destruction with respect to conversional acid-catalyzed hydrolysis is given in Table 2. In contrast to conventionally used hydrolysis techniques the EBP-stimulated processing is rapid single-stage, and environmental friendly procedure. Often the stable *N*-acetyl group presented in chitin structure is required for further (bio)chemical modifications of chitooligosaccharides. The chemical hydrolysis of chitin resulted in the formation of significantly deacetylated LMWP whereas the intensive deacetylation of chitin oligomers does not occur during the EBP-treatment.

The advantages of EBP-treatment with respect to gas discharge plasmas have been discussed in our previous papers [17,18,19]. Furthermore, the effective controllable destruction of polysaccharides occurs due to the EBP-hydrolysis, whereas the treatment in gas discharge plasmas mostly results in biomaterial oxidation and an improvement of its hydrophilic properties [42,43,44].

## 4. Materials and Methods

High molecular weight crab shell chitin (viscosity-average molecular weight, *M_ν_* = 1000 kDa) obtained from red king crab *Paralithodes camtschaticus*, cellulose sulfate (molecular weight 160 kDa), and microcrystalline cellulose with molecular weight of 55 kDa were used as the original substances for the further EBP-treatment. The chitin was of high purity and free of any other biopolymers/inorganic content and pigments (content of proteins and other impurities <0.5%). The substance was obtained from Bioprogress Co., (Shchelkovo, Russia) and certified by the manufacturer. All substances were water-insoluble white powders.

For solubility measurements, 100 ± 0.1 mg of the preliminary dried sample (*m_s_*) were placed into a tube and 1.5 mL of distilled water or 5% NaOH (Sigma-Aldrich, Munich, Germany) was added to the sample. The resulting mixture was incubated for 24 h at room temperature under periodic mixing. After the incubation the mixture was centrifuged for 5 min and 1 mL of centrifugate was taken and dried. The mass of the dry residue (*m_dr_*) was measured with an accuracy ±0.1 mg. The sample solubility was calculated as the (*m_dr_*/*m_s_*) × 100% ratio.

Molecular masses (weight-average *M_w_*, number-average *M_n_*, and *z*-average mass *M_z_*) of the chitin EBPtreatment products were determined by the size exclusion chromatography on a LC-20 Prominence HPLC system (Shimadzu, Tokyo, Japan) equipped with refractometric detector RID-10A. The chromatographic column was Ultrahydrogel 500 7.8 × 300 mm (Waters, Milford, MA, USA). Other analysis conditions were as follows:the mobile phase—buffer containing 0.05 M acetic acid and 0.15 M ammonium acetate (pH = 5.1); the flow rate—0.5 mL/min; temperature—30 °C; volume of the injected sample—40 μL; duration of the analysis—25 min. Dextrans (Sigma, St. Louis, MO, USA) were used as standard samples for mass scale calibration.

Individual *N*-acetylglucosamine oligomers were determined by the reverse phase chromatography using the Asahipak NH2P-LF (Shodex, Tokyo, Japan) chromatographic column. The column was calibrated with a model mixture of *N*-acetylglucosamine oligomers with the polymerization degree *n* = 1–6.

The size exclusion chromatography of cellulose materials was performed using the chromatograph Staier (Akvilon, Moscow, Russia) and the chromatographic column Phenomenex BioSep-Sec-S-3000 (Phenomenex, Torrance, CA, USA) with the efficiency of 30,000 theoretical plates. The analysis conditions were as follows:the elutriating agent—0.1 M phosphate buffer (pH 6.86) containing 0.05% NaN_3_; the elution rate—1 mL/min; temperature—30 °C; UV-detector with the wavelength 280 nm. The correlation between elution time (*t*) and molecular mass of cellulose oligosaccharides (M) is described by Equation (2):

ln(М) = −0.193*t*^2^ + 1.91*t* + 9, (2)

where (*t* = 6–11 min); the validity coefficient of approximation *R*^2^ = 0.99. After *t* = 11 min the column operates by the reversed-phase mechanism.

The ligand-exchange high-performance chromatography of cellulose products was performed at 30 °C under the following conditions: ion-exclusion column Rezex RSO-Oligosaccharide Ag^+^ (4%) (Phenomenex, Torrance, CA, USA) with the efficiency of 12,000 theoretical plates; the elutriating agent—H_2_O; the elution rate—600 mcl/min. Differential refractometric detection (Water, Milford, CT, USA) was applied.

Solutions from the original and the EBP-treated chitosan (*M_ν_* = 500 kDa with the degree of deacetylation 85–98% and polydispersity index 1.5) were prepared by dissolving them in 1% acetic acid and the distilled water respectively. The solutions were cast and dried at 37 °C to form thin films 8.0 ± 1.5 μm thick. The obtained films were then analyzed by Fourier transform infrared (FT-IR) spectroscopy. In particular, the film thickness was estimated by analyzing a baseline modulation period in a transmittance spectrum of the chitosan film.

IR spectral measurements in a wavenumber (ν) range 500–5000 cm^−1^ with a resolution of 4 cm^−1^ were carried out by a FT-IR spectrometer Perkin-Elmer Spectrum 100 (Perkin-Elmer, Boston, MA, USA). A transmittance spectrum of the studied chitosan or cellulose film, *T*(ν), was measured at normal incidence of IR radiation on the film surface as a ratio:

*T*(ν) = *T*_1_(ν)/*T*_0_(ν), (3)

where *T*_0_(ν) and *T*_1_(ν) are the registered transmission spectra of the spectrometer channel for an empty sample holder and for a sample holder with the film, respectively. To get acceptable signal-to-noise ratio 16 scans were statistically averaged. The transmittance spectrum was converted to obtain the absorbance spectrum of the film, *A*(ν), through Equation (4):

*A*(ν) = −log_10_(*T*_1_(ν)/*T*_0_(ν)). (4)

The cellulose crystallinity index was determined by means of X-ray diffraction analysis (XRD) using the method developed by Segal and coworkers [45].

**Figure 12:**
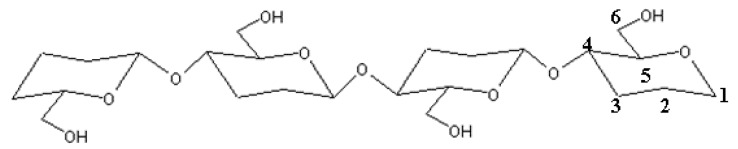
The structure of the model β(1→4)-tetramer.

**Figure 13:**
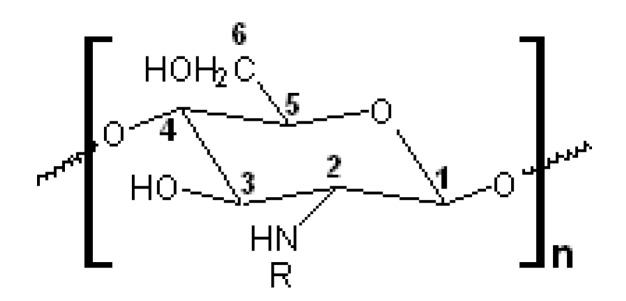
The scheme of the protons in the chitin monomer.

^1^H- and ^13^C-NMR spectra of the EBP-produced cellulose and chitin oligosaccharides were recorded on a Bruker Avance 500 MHz instrument (Bruker, Billerica, MA, USA) at 25 °C using a standard 5-mm probe from 150 mg of sample dissolved in 1.0 mL D_2_O after 10,000 scans. The ^13^C-NMR spectra of the EBP-treated cellulose were obtained under total proton decoupled conditions and the model β(1→4)-tetramer (Figure 12) was used as a reference substance. The average degree of the EBP-treated chitin acetylation (DA, mol %) and deacetylation (DD, mol %)were determined according to Equation (5) [46]:

DD (mol %) = {1 − [1/3I_CH_3__/(1/6I_H_2_–H_6__)]}100, (5)

where I_CH_3__—the integral intensity of CH_3_ residue and I_H_2_–H_6__—the sum of the integral intensities of I_H_2__, I_H_3__, I_H_4__, I_H_5__, I_H_6__ and I_H_6__’ protons (Figure 13).

## 5. Conclusions

1. The possibility of the EBP-stimulated degradation of chitin and cellulose and formation of the water-soluble oligosaccharides was proved experimentally.
2. Chitooligosaccharides with weight-average molecular mass 1000–2000 Da and polydispersion index 2.2 and cellulose oligosaccharides with polymerization degrees 3–10 were formed due to the destruction of β-1,4-glycosidic bonds during the EBP-treatment. The EBP-processing resulted in 25–40% deacetylation of original chitin and partial oxidation of the produced oligosaccharides.
3. At optimized treatment conditions (plasma generating media pressure, electron beam characteristics, design of the mixing device) 85% yield of low-molecular weight COS and 77.4% yield of cellulose oligosaccharides were obtained in only 2 and 10 min, respectively.
4. Both the fast electrons and the active oxygen particles produced in the EBP are responsible for the destruction of β-1,4-glycosidic bonds in the original polysaccharides. By proper selection of treatment conditions, the polysaccharide damage due to high-energy electrons can be minimized and the plasma chemical processes predominate.

## Figures and Tables

**Table 1:** The solubility changes of the EBP-treated cellulose sulfate in distilled water and 5% NaOH.

Time of EBP-Processing, min	Yield of Water-Soluble Products, %	Yield of Products, Soluble in 5% NaOH, %	Yield of Non-Soluble Residue ^1^, %	Polymerization Degree of Non-Soluble Residue
0	1.8	6.8	93.2	960
2	22.7	45.3	54.7	460
10	77.4	100	0	-

^1^ Non-soluble residue corresponds to products that do not dissolve in either 5% NaOH or distilled water.

**Table 2:** The comparison of the EBP-stimulated polysaccharides destruction with respect to acid-catalyzed chemical hydrolysis.

Criteria	Chemical Hydrolysis	EBP-Processing
Treatment time	Several hours or days	Minutes (τ = 2–10)
Number of stages	Multi-stage	Single-stage
Specificity and Efficiency	Destruction of β-1,4-glycosidic bonds; Destruction of amorphous parts of biopolymer predominates; Both oxidation changes of produced oligosaccharides and deep oxidation of original biopolymers are possible.	Destruction of β-1,4-glycosidic bounds; Destruction of both amorphous and crystalline parts of biopolymer occurs; Oxidation changes of oligosaccharides (formation of C=O and –COOH groups).
Chitin deacetylation	Deacetylated low molecular weight derivatives of chitin are produced. Reacetylation is needed	The DD of produced chitin oligosaccharides 25–40%.
Molecular mass of products	Cellulose fragments with polymerization degree 200 or monomers are produced [40]	LMWP from dimers to heptamers are produced.
LMWP yields	Low yields of LMWP and large amounts of monomeric units [40,41].	Up to 80–85%
Ecological safety	Highly concentrated acidic or alkaline solutions are needed. Toxic wastes are produced. High energy consumption.	Environmentally friendly: hazardous byproducts and toxic wastes are not generated.
